# Risk of death after first-time blood stream infection in incident dialysis patients with specific consideration on vascular access and comorbidity

**DOI:** 10.1186/s12879-018-3594-7

**Published:** 2018-12-20

**Authors:** Karl Emil Nelveg-Kristensen, Gunnar Hellmund Laier, James Goya Heaf

**Affiliations:** 1 Department of Nephrology, Rigshospitalet, Copenhagen University Hospital, Copenhagen, Denmark; 2 Department of Production, Research and Innovation, Region zealand, Sorø, Denmark; 3 Department of Medicine, Zealand University Hospital, Roskilde, Denmark

**Keywords:** Dialysis, Blood stream infection, Risk factors, Dialysis modality, Vascular access

## Abstract

**Background:**

The mortality following blood stream infection (BSI) and risk of subsequent BSI in relation to dialysis modality, vascular access, and other potential risk factors has received relatively little attention. Consequently, we assessed these matters in a retrospective cohort study, by use of the Danish nation-wide registries.

**Methods:**

Patients more than 17 years of age, who initiated dialysis between 1.1.2010 and 1.1.2014, were grouped according to their dialysis modality and vascular access. Survival was modeled in time-dependent Cox proportional hazard analyses. Potential risk factors confined by a modified Charlson comorbidity index (MCCI), were subsequently assessed in stepwise selection models.

**Results:**

At baseline, 764 patients received peritoneal dialysis (PD), and 434, 479, and 782 hemodialysis (HD) patients were dialyzed by use of arteriovenous fistulas (AVFs), tunneled catheters (TCs), and non-tunneled catheters (NTCs), respectively. We identified 1069 BSIs with an overall incidence rate of 17.7 episodes per 100 person years, and 216 BSIs occurred more than one time in the same patient. HRs of post BSI mortality relative to PD were 3.20 (95% CI 1.86–5.50; *p* < 0.001) with NTCs; whereas no associations were found for AVF and TC. The risk of subsequent BSIs was higher with NTCs [HR 2.29 (95% CI 1.09–4.82), *p* = 0.030], and no significant difference was found for AVF and TC, in relation to PD. There was an increased risk of both outcomes with TC relative to AVF [death: 1.57 (95% CI 1.07–2.29, *P* < 0.021); BSI: 1.78 (95% CI 1.13–2.83, *P* < 0.014], and risk of death was reduced in patients who changed to AVF after first-time BSI. The MCCI was significantly associated with the risk of subsequent BSI and post BSI death; however, only some of the variables contained in the index were found to be significant risk predictors when analyzed in the fitted model.

**Conclusions:**

While NTC was the most predominant risk factor for subsequent BSI and post BSI mortality, AVF appeared protective.

**Electronic supplementary material:**

The online version of this article (10.1186/s12879-018-3594-7) contains supplementary material, which is available to authorized users.

## Background

Approximately 10% of the total global population is affected by chronic kidney disease (CKD), and over two million people receive dialysis worldwide, notwithstanding that this amount only represents a fraction (10%) of the people who need renal replacement therapy (RRT) to stay alive [1, 2]. Blood stream infection (BSI) is a significant threat among patients receiving RRT and remains the second most frequent cause of death in this population [3, 4]. Thus, the risk of BSI was recently found to be 28 times greater in patients receiving hemodialysis (HD) than in a matched population without kidney disease [5].

There are several contributing factors to this high frequency of BSI, e.g., advanced comorbidity, immune dysfunction caused by uremia, and frequent disruptions of the natural skin barrier [6–8]. Some of these factors such as RRT modality and vascular access type can be directly modified by health care professionals and thus supply a potential opportunity for interventions that eventually can reduce the risk of BSI and subsequent post BSI mortality. In this regard, patients receiving peritoneal dialysis (PD) or HD via non-catheter-based access types, i.e., arteriovenous fistulas (AVFs) and arteriovenous grafts (AVGs), have a much lower incidence of BSI than patients receiving HD by use of central venous catheters, i.e., tunneled central venous HD catheters (TCs) and non-tunneled catheters (NTCs), respectively, in which the latter has been associated with the highest incidence rates [7, 9–15]. Accordingly, such risk factors that are susceptible for improvements deserve study.

Numerous studies have examined the epidemiology of BSI, particularly in HD patients. However, the mortality following BSI has until now received relatively little attention, and there has been a preferential focus on infections caused by *Staphylococcus aureus (S. aureus)* [7, 16–19]. Large scale studies are generally hampered by the lack of specific patient data, while small, center-based studies have insufficient statistical power. The establishment in 2010 of the Danish Microbiology Database (MiBa) now allows detailed examination of the epidemiology, potential risk factors, and prognosis of BSI amongst patients receiving dialysis. Consequently, in the current study, using Danish nationwide registries, we aim to describe the incidence of BSI and the resulting causal microbial species, in an incident cohort of RRT patients, with specific consideration of HD and PD, as well as subsequent access modalities of HD, i.e., TC, NTC, and AVF, respectively. Furthermore, we will examine the risk of subsequent BSI episodes and post BSI mortality among patients in HD, with explicit focus on HD access modality and risk factors confined in a modified Charlson comorbidy index.

## Methods

### Patients and registries

This study was prepared as a retrospective, epidemiologic cohort study concerning all patients more than 17 years of age, who initiated dialysis (PD or HD) between 1.1.2010 and 1.1.2014, using the Danish Nephrology Registry (DNR), MiBa, and the Danish nationwide administrative registries as data source.

All citizens in Denmark have a unique personal identification number, which allows for individual-level linkage of information from the applied registries. DNR was established in 1990 and contains comprehensive data concerning all patients receiving RRT [20]. MiBa contains nationwide data on microbiological diagnoses, including all positive blood cultures identified in Denmark since 2010 [21]. Since 1978, information on all admissions to Danish hospitals, according to the international classification code (ICD) and the Nordic classification of surgical procedures (NCSP), respectively, have been registered in the National Patients Registry [22, 23]. Information on age, sex, renal diagnosis, date of referral to a nephrology department, date of dialysis initiation, access type at first access, and date of RRT modality changes (HD, PD or transplantation (RT)) was obtained from the DNR. This registry also supplied information on initial and subsequent dialysis modality and access type, i.e., PD, AVF, NTC and TC. AVGs are not regularly used in Denmark. Date of all surgical TC and AVF procedures, changes from one vascular access type to another, and information on comorbidity at the day of dialysis initiation were obtained from the National Patient Registry. The primary cause of death was obtained from the DNR. We applied a modified Charlson comorbidity index (MCCI), in which each of the following comorbidities was assigned one index point: diabetes (DM), heart failure, ischemic heart disease, peripheral vascular disease, ischemic/hemorrhagic/neurologic CNS disease, chronic obstructive pulmonary disease (COPD), collagenoses, gastric ulcer, liver disease, and cancer (all types, including leukemia). All patients were automatically allocated one index point for CKD. The index was further divided in three levels, i.e. MCCI = 1, MCCI = 2–3 and MCCI > 3 for use in the main statistical model.

The date and result of all positive cultures for the included patients were obtained from MiBa. In specific situations with unreproducible positive cultivations of e.g. opportunistic skin-associated low virulence microorganisms and lack of clinically signs of BSI, positive cultures were registered as ‘possible contamination’ by the corresponding microbiologic departments. Positive cultures identified before the date of first dialysis were excluded. Multiple positive cultures on the same day were classified as polymicrobial. Multiple positive cultures within a 7-day period were regarded as one BSI episode, with the first positive culture being registered as the causative agent. Bacterial diagnoses were classified into 13 major groups for analysis: *S. aureus*, Coagulase Negative Staphylococci (CNS), Unspecified Staphylococci (where only the genus was reported, and not the species), Streptococci, Enterococci, Other gram-positive bacteria, *Escherichia coli* (*E. coli*), Enterobacter, *Klebsiella pneumoniae* (*klebsiella*)*, Pseudomonas aeruginosa* (*pseudomonas*), Other gram-negative bacteria, Fungi, and Other (where a positive blood cultivation was reported without genus or species). In the current study, CNS was defined as the ability of the staphylococcus species to coagulate plasma, which basically included all other Staphylococci than *S. aureus*. For a full presentation of the microorganisms included in each group, please see Additional file 1.

PermCath catheters are the standard cuffed catheters of choice in Denmark. Routinely, TCs are maintained by use of aseptic techniques applied by adequately trained personnel. In the setting of BSI, TCs are in general temporarily removed in favor of a NTC, and a new TC is inserted when the infection has resolved. In the absence infectious involvement of the tunnel, TCs may be renewed by guidewire catheter exchange, and in some settings, the TC is left in place, depending on infection severity, microbiology, and vascular accessibility, however, antibiotic lock solutions are not commonplace in Denmark. In terms of AVFs, these are usually allowed a ripening period of approximately 6 weeks before usage, and buttonhole needling is the most frequently applied method for cannulation; although, rope ladder needling may be preferred in some departments. Overall, to cover a wide spectrum of both gram-positive and gram-negative causative organisms in vascular access-related BSIs, and due to the relatively low occurrence of methicillin resistant *Staphylococcus aureus* in Denmark, a broad-spectrum beta-lactam antibiotic agent is utilized as first choice empirical treatment, e.g., piperacillin with tazobactam or cefuroxime. The antibiotic therapy is subsequently tailored according to the microbiologic cultivation results.

### Statistics

Crude incidence rates (IRs) for each study outcome per 100 person-years were calculated for patients receiving PD and HD, as well as each HD vascular access type. Differences in baseline characteristics were analyzed with Chi-square test for categorical covariates and Student’s t-test or the non-parametric Kruskal-Wallis and Mann-Whitney tests for continuous variables, respectively. Time dependent Cox proportional hazard models were used to model survival and other endpoints, and outcomes were expressed as hazard ratios (HRs). Patients entered the model at the day of dialysis initiation, and the cohort was followed until occurrence of a study outcome or until 1.1.2015. The following outcomes were assessed in different models: BSI, all-cause mortality, subsequent BSIs (after first-time BSI) and post BSI mortality. In analyses of all-cause mortality and first-time BSI, each observation was split at any change in dialysis modality or vascular access, so that these variables could change time-dependently. In analyses on the effect of first-time BSI in risk of all-cause mortality as compared to patients without BSI, each observation was also split at the day the causal microorganism was isolated, to avoid immortal time bias. In the absence of registered TC or AVF creation, all NTCs were censored 90 days after initiation. In addition, all analyses were adjusted for age, sex, calendar year, MCCI, and Gram stain. The latter was only included in consideration of patients who have had a first-time BSI. Results were tested in sensitivity analyses where all covariates were included at baseline. Furthermore, we modeled the risk of all-cause mortality and first-time BSI in baseline analyses where a 60-day qualifying period, i.e., the date of dialysis initiation (index date) plus 60 days, was added to avoid an anticipated high risk of complications in close temporal proximity to RRT initiation. As we did not have information on the specific infectious focus preceding a BSI, we performed a baseline subgroup analysis of patients with first-time BSI caused by *S. aureus,* which are most likely to be caused by the vascular/peritoneal access. Also, we investigated the effect of vascular access change subsequent to first-time BSI on risk of post BSI mortality. Time-dependent analyses were censored at the day of RT, and change of modality. Baseline analyses were additionally censored at the day of change in vascular access. Finally, each variable confined by the MCCI was evaluated in a stepwise selection model with alternating forward selection and backward elimination, i.e., only variables with a univariate *P* value < 0.25 were allowed to be tested further in the stepwise model, and only variables that contributed with a *P* value of < 0.15 were contained in the fitted model. The proportional hazard assumption, linearity of continuous variables and absence of interaction between variables were fulfilled if not otherwise stated. A two-sided *P* value of 0.05 or less was considered significant. *P* values > 0.10 are denoted in the text as ‘not significant’ (NS) unless stated otherwise. Analyses and data management were performed in SAS version 9.4 (SAS Institute Inc. Cary, North Carolina) and R version 3.3.3 (R Foundation for Statistical Computing, Vienna, Austria).

## Results

### Population

In the period January 2010–January 2014, 2646 incident men and women more than 17 years of age, receiving RRT were identified from DNR, of whom 1742 received HD, 764 PD and 140 underwent RT as their first registered RRT modality, leaving 2506 patient ready for inclusion. At baseline, 434, 479, and 782 HD patients were dialyzed by use of AVFs, TCs, and NTCs, respectively. Forty-seven patients did not have their initial HD access registered. Patients with missing data concerning initial access did not differ significantly from the other patients (data not shown). During follow up, 1027 patients died of which 366 occurred after BSI, 303 underwent RT, and 124 regained some degree of native renal function. The mean age at baseline in the total study population was 64.8 ± 15.2 years, with an overall overweight of male gender (65.4%). In general, patients who initially received a NTC were of the same age; however, they were burdened with excess comorbidity as compared to patients with AVF, TC and PD. Conversely, the group of patients who started on PD were slightly younger, and presented with a lower percentage of patients classified with high MCCI scores. Other baseline characteristics are shown in Table 1. Mean follow up for the total study population was 1.7 ± 1.4 (median 1.5, interquartile range (IQR) 0.3–2.7) years.

**Table 1:** Baseline characteristics of incident patients receiving renal replacement therapy during 2010–2013 with specific consideration on peritoneal dialysis and hemodialysis vascular access type

	PD	AVF	TC	NTC	*P* value
N	764	434	479	782	
Male (%)	509 (66.6)	289 (66.6)	296 (61.8)	514 (65.7)	NS
Age (mean (SD))	63.3 (14.8)	65.9 (14.0)	65.0 (16.4)	65.5 (15.3)	0.025
MCCI (%)					< 0.001
2	225 (29.5)	92 (21.2)	84 (17.5)	119 (15.2)	
3	127 (16.6)	77 (17.7)	66 (13.8)	108 (13.8)	
4	173 (22.6)	85 (19.6)	96 (20.0)	134 (17.1)	
5	103 (13.5)	66 (15.2)	93 (19.4)	150 (19.2)	
> 5	133 (17.4)	114 (26.3)	138 (28.8)	265 (33.9)	
Missing	3 (0.4)	0 (0.0)	2 (0.4)	6 (0.8)	
Average MCCI	3.9 (1.8)	4.4 (2.1)	4.7 (2.2)	4.9 (2.3)	
Median (IQR)	4 (2–5)	4 (3–6)	4 (3–6)	5 (3–6)	
Renal diagnosis (%)					< 0.001
CIN	25 (3.3)	20 (4.6)	18 (3.8)	31 (4.0)	
DM	185 (24.2)	111 (25.6)	134 (28.0)	202 (25.8)	
GN	90 (11.8)	26 (6.0)	46 (9.6)	60 (7.7)	
Hypertension	103 (13.5)	71 (16.4)	56 (11.7)	96 (12.3)	
PKD	58 (7.6)	48 (11.1)	25 (5.2)	19 (2.4)	
Other	74 (9.7)	55 (12.7)	86 (18.0)	194 (24.8)	
Unknown	229 (30.0)	103 (23.7)	114 (23.8)	180 (23.0)	
BSI episodes					NS
1	123 (79.9)	90 (73.2)	119 (68.4)	224 (70.9)	
2	28 (18.2)	23 (18.7)	38 (21.8)	76 (24.1)	
3	1 (0.6)	8 (6.5)	14 (8.0)	10 (3.2)	
4	1 (0.6)	1 (0.8)	2 (1.1)	3 (0.9)	
5	1 (0.6)	1 (0.8)	1 (0.6)	2 (0.6)	
6	0 (0.0)	0 (0.0)	0 (0.0)	1 (0.3)	
Microorganisms (%)					NS
*S. aureus*	31 (20.1)	42 (34.1)	70 (40.2)	102 (32.3)	
*E. coli*	30 (19.5)	15 (12.2)	15 (8.6)	42 (13.3)	
Enterococci	11 (7.1)	7 (5.7)	10 (5.7)	28 (8.9)	
Enterobacter	5 (3.2)	5 (4.1	14 (8.0)	9 (2.8)	
Other gram-neg	12 (7.8)	4 (3.3)	9 (5.2)	17 (5.4)	
Other gram-pos	4 (2.6)	7 (5.7)	4 (2.3)	15 (4.7)	
*Klebsiella pneumoniae*	8 (5.2)	10 (8.1)	5 (2.9)	16 (5.1)	
*Pseudomonas aeruginosa*	3 (1.9)	1 (0.8)	6 (3.4)	7 (2.2)	
CNS^a^	22 (14.3)	13 (10.6)	15 (8.6)	40 (12.7)	
Unspecified staphylococci^b^	15 (9.7)	11 (8.9)	12 (6.9)	25 (7.9)	
Streptococci	10 (6.5)	6 (4.9)	9 (5.2)	12 (3.8)	
Fungi	3 (1.9)	0 (0.0)	2 (1.1)	2 (0.6)	
Other^b^	0 (0.0)	2 (1.6)	3 (1.7)	1 (0.3)	

*PD* peritoneal dialysis, *AVF* arteriovenous fistula, *TC* tunneled catheter, *NTC* non-tunneled catheter, *MCCI* Modified Charlson comorbidity index, *CIN* chronic interstitial nephritis, *DM* diabetes mellitus, *GN* glomerulonephritis, *PKD* polycystic kidney disease, *NS* not significant, *CNS* Coagulase Negative Staphylococci. For a full disclosure of microorganisms included in each of the 13 microbial groups outlined in this table, see Additional file 1. The *P* value is calculated as the overall difference between groups or the overall difference of distribution between groups

^a^ Defined as the ability of the organism to coagulate plasma, i.e. all other Staphylococci than S. aureus

^b^ Only the genus was reported, not the species

^c^ A positive blood cultivation was reported without genus or species

**Fig. 1:**
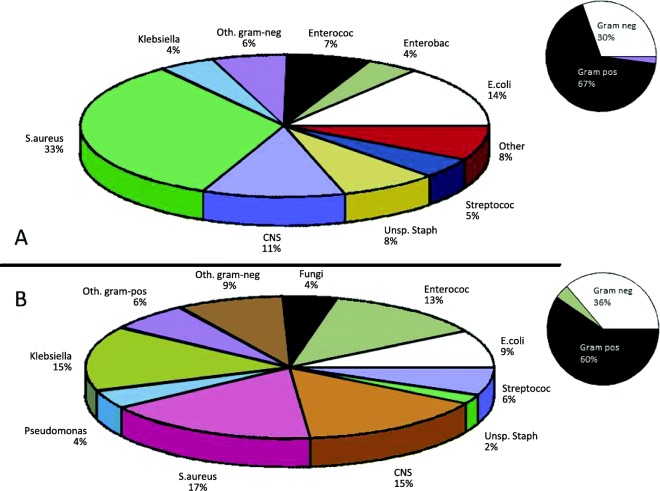
Distribution of causal microbiologic isolates at first-time BSI (**a**) and after more than two BSI episodes (**b**). CNS: coagulase negative staphylococci. The *P*-value for overall difference in distribution of microbiologic isolates between **a** and **b** were < 0.001

We identified 1069 episodes of bacteremia in 784 patients corresponding to an overall IR of 17.7 episodes per 100 person years. A total of 169, 33, and 14 bacteremia episodes occurred two, three and more than three times in the same person. In 71 cases, the putative causative microorganism was registered as ‘possible contamination’ and 30 cases were classified as polymicrobial. With *S. aureus* (32.1%) and *E. coli* (13.3%) being the primary individual isolates, the overall Gram-positive and Gram-negative bacteria accounted for 68.0 and 29.7% of the cultured microorganisms, respectively. *E. coli* was isolated in 19.5% of the bacteremia episodes among patients on PD, and in 11.8% among those on HD. Conversely, *S. aureus* appeared more frequently among HD patients as compared with PD (35.1% vs. 20.1%). As shown in Fig. 1, the composition of microorganisms in the first encountered bacteremia differed considerably from what was isolated after more than two bloodstream infections, with a slight reduction in Gram positive microorganisms. Where first-time bloodstream infections were dominated by *S. aureus* and *E. coli*; fungi, other Staphylococcus species, Enterococci, *Klebsiella* and *Pseudomonas* were significantly more prevalent among patients with more than two blood stream infections (Fig. 1). The overall distribution of the 13 pre-specified microbiologic groups for first-time BSI according to the vascular access modality as well as PD are shown in Table 1.

### Survival analyses

**Fig. 2:**
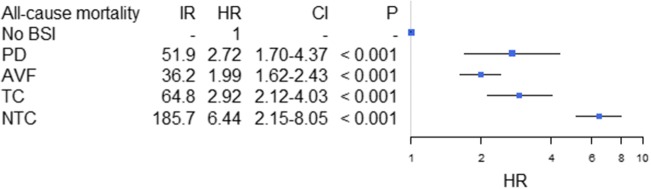
Risk of post BSI mortality in patients with a first-time BSI. Results from the adjusted time-dependent Cox proportional hazard analyses. IR: Incidence rate per 100 patient-years; HR: Hazard ratio for the risk of death after first time BSI. Patients without BSI are used as reference. AVF: arteriovenous fistula; TC: tunneled catheter; NTC: non-tunneled catheter; BSI: blood stream infection. Error bars illustrate 95% confidence intervals

**Fig. 3:**
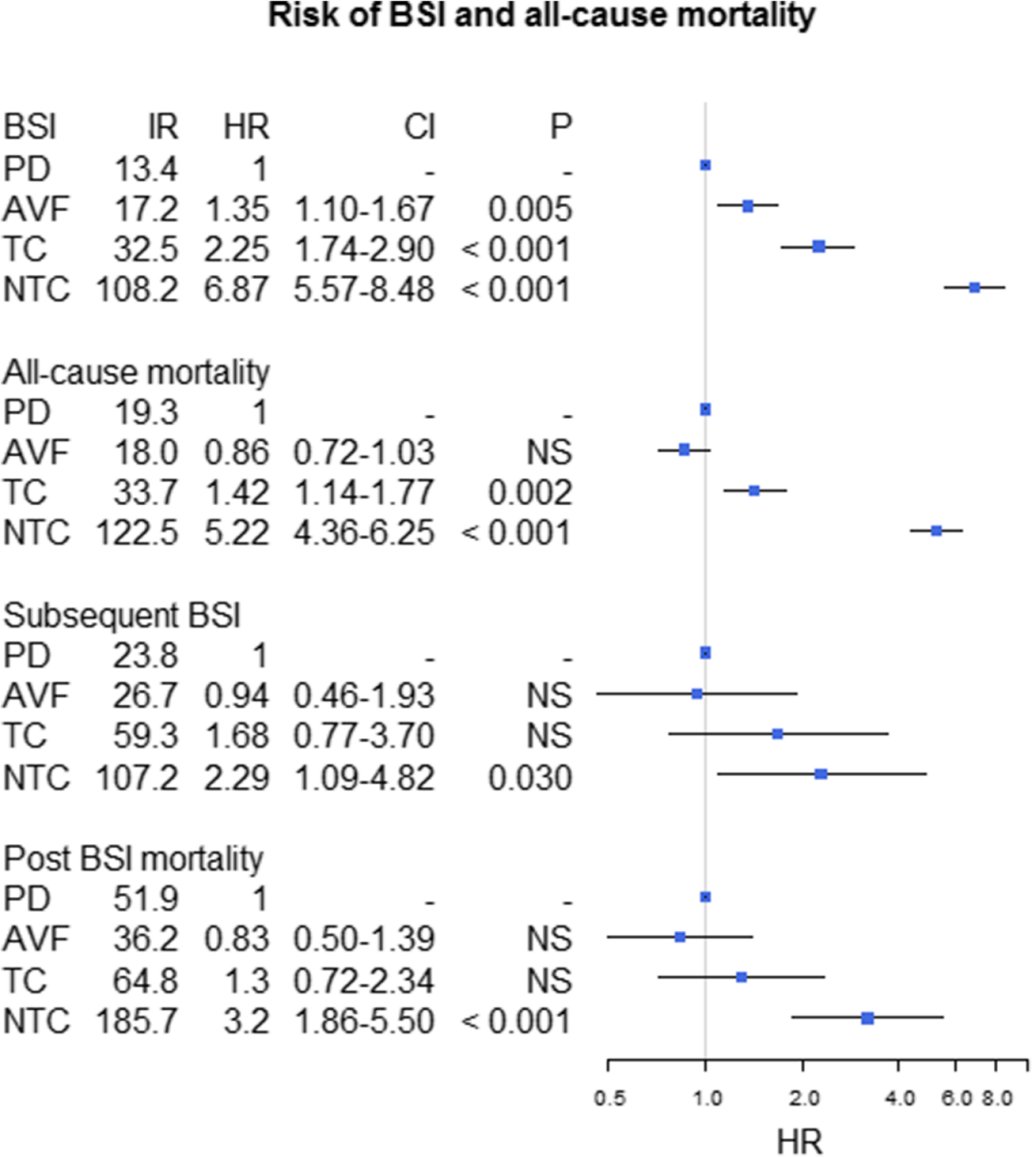
Risk of blood stream infection (BSI) and death in the total study population and after first-time BSI. Results from the adjusted time-dependent Cox proportional hazard analyses. IR: Incidence rate per 100 patient-years; HR: Hazard ratio for the risk of BSI and all-cause mortality, as well as subsequent BSI episodes and post BSI mortality after first-time BSI. Patients receiving peritoneal dialysis (PD) are used as reference. AVF: arteriovenous fistula; TC: tunneled catheter; NTC: non-tunneled catheter; BSI: blood stream infection. Error bars illustrate 95% confidence intervals

Crude IRs for first-time BSI and all-cause mortality are shown in Table 2. Censored IRs are listed in Figs. 2 and 3. In the fully adjusted time-dependent Cox proportional hazard analyses we found an increased risk of BSI for patients receiving HD by use of AVF [HR 1.35 (95% CI 1.10–1.67), *P* = 0.005], TC [HR 2.25 (95% CI 1.74–2.90), *P* < 0.001], and NTC [HR 6.87 (95% CI 5.57–8.48), *P* < 0.001] relative to patients receiving PD (Fig. 3). Also, there was an increased risk of all-cause mortality associated with TC and NTC with HRs of 1.42 (95% CI 1.14–1.77, *P* = 0.002) and 5.22 (95% CI 4.36–6.25, *P* < 0.001), respectively. However, there was no significant difference in risk of all-cause mortality for AVF [HR 0.86 (95% CI 0.72–1.03), NS] relative to PD (Fig. 3). Comparable results were found in baseline analyses where patients entered the model at the date of dialysis initiation, and when a 60-day qualifying period was applied (Additional file 2).

**Table 2:** Crude incidence rates for first-time blood stream infection and all-cause mortality

Modality/Access	N	Risk time^a^	IR^b^	Interval	N	Risk time^a^	IR^b^	Interval
	Blood stream infection				All-cause mortality			
Total	784	44.2	17.7	16.5–19.0	1027	44.2	23.2	21.8–24.7
PD	154	16.3	9.43	8.05–11.0	227	16.3	13.9	12.2–15.8
HD	630	27.9	22.6	20.9–24.4	800	27.9	28.7	26.7–30.7
- AVF	123	9.78	12.6	10.5–15.0	153	9.78	15.6	13.3–18.3
- TC	174	9.40	18.5	16.0–21.5	219	9.40	23.3	20.4–26.6
- NTC	316	7.9	39.8	35.6–44.4	407	7.9	51.2	46.5–56.46

*PD* peritoneal dialysis, *HD* hemodialysis, *AVF* arteriovenous fistula, *TC* tunneled catheter, *NTC* non-tunneled catheter, *IR* incidence ratio. ^a^ 10^2^ patient years. ^b^ 1/100 patient years

In time-dependent Cox analyses modeled to examine the risk of death after first-time BSI as compared to all patients without BSI, regardless of dialysis and vascular access type, we found a significantly increased risk of death associated with all modalities with HRs of 2.72 (95% CI 1.70–4.37, *P* < 0.001), 1.99 (95% CI 1.62–2.43, *P* < 0.001), 2.92 (95% CI 2.12–4.03, *P* < 0.001), and 6.44 (95% CI 5.15–8.08, *P* < 0.001) for PD, AVF, TC and NTC, respectively (Fig. 2). Confining the analysis to only include patients after their first-time BSI with PD as reference, there was no significant difference between AVF and TC relative to PD; whereas NTC remained significantly associated with a marked increase in risk of death (Fig. 3). Comparable results were found in analyses based on baseline variables, as well as in the subgroup analyses that only included patients with a first-time BSI caused by *S. aureus* (Additional file 2). Also, the risk of subsequent BSI episodes was significantly higher in HD patients who encountered their first BSI with a NTC [HR 2.29 (95% CI 1.09–4.82), *p* = 0.030], whereas no significant difference was found for patients with AVF [HR 0.94 (95% CI 0.46–1.93), NS] and TC [HR 1.68 (95% CI 0.77–3.70), NS]. In post hoc analyses, HRs for post BSI mortality and subsequent BSI with TCs, using AVF as reference, were 1.57 (95% CI 1.07–2.29, *P* < 0.021) and 1.78 (95% CI 1.13–2.83, *P* < 0.014), respectively. Finally, we found an overall decreased risk of all-cause mortality associated with patients who changed their vascular access in the period after first BSI episode [HR 0.61 (95% CI 0.47–0.79), *P* < 0.001]; however, this association was not evident in a subgroup analysis restricted to those who did not receive an AVF [HR 0.73 (95% CI 0.41–1.23), NS]. Estimates regarding the other included covariates in the main time-dependent Cox models, and from the two-step fitted model covering each of the covariates included in the modified comorbidity score, are shown in Tables 3 and 4, respectively.

**Table 3:** Risk of blood stream infections and mortality associated with covariates included in the time-dependent cox proportional hazard models

Model entry	variable	HR (CI)	*P* value	HR (CI)	*P* value
		Blood stream infection		Mortality	
RRT initiation	AVF^a^	1.35 (1.20–1.67)	0.005	0.86 (0.72–1.03)	NS
	TC^a^	2.25 (1.74–2.90)	< 0.001	1.42 (1.14–1.77)	0.002
	NTC^a^	6.87(5.53-8.48)	< 0.001	5.22 (4.36–6.25)	< 0.001
	Sex	1.10 (0.95–1.23)	NS	1.02 (0.89–1.16)	NS
	Age 50–59^b^	1.19 (0.92–1.54)	NS	1.55 (1.10–2.18)	0.012
	Age 60–69^b^	1.05 (0.83–1.32)	NS	2.14 (1.59–2.90)	< 0.001
	Age > 70^b^	0.91 (0.73–1.13)	NS	3.71 (2.79–4.93)	< 0.001
	MCCI (2, 3)^c^	0.87 (0.71–1.08)	NS	1.75 (1.36–2.24)	< 0.001
	MCCI (> 3)^c^	1.14 (0.94–1.40)	NS	2.76 (2.18–3.51)	< 0.001
Post BSI	AVF^a^	0.94 (0.46–1.93)	NS	0.83 (0.50–1.39)	NS
	TC^a^	1.68 (0.77–3.70)	NS	1.30 (0.72–2.34)	NS
	NTC^a^	2.29 (1.09–4.82)	0.030	3.20 (1.86–5.50)	< 0.001
	Sex	1.14 (0.81–1.60)	NS	1.07 (0.83–1.38)	NS
	Age 50–59^b^	1.47 (0.85–2.56)	NS	1.53 (0.82–2.86)	NS
	Age 60–69^b^	1.25 (0.75–2.10)	NS	2.56 (1.50–4.37)	< 0.001
	Age > 70^c^	1.35 (0.81–2.23)	NS	5.41 (3.26–9.00)	< 0.001
	MCCI (2, 3)^c^	1.70 (0.94–3.07)	0.081	1.96 (1.20–3.20)	0.008
	MCCI (> 3)^c^	2.99 (1.72–5.19)	< 0.001	2.99 (1.88–4.74)	< 0.001
	Gram +	0.89 (0.62–1.27)	NS	0.77 (0.59–0.99)	0.044

*HD* hemodialysis, *AVF* arteriovenous fistula, *TC* tunneled catheter, *NTC* non-tunneled catheter, *MCCI* modified Charlson comorbidity index, *NS* not significant, *HR* hazard ratio, *CI* confidence interval

^a^ Analyzed as a four-leveled variable with peritoneal dialysis as reference

^b^ Analyzed as a four-leveled variable with ‘age < 50 years’ as reference

^c^ Analyzed as a three-leveled variable with ‘MCCI = 1’ as reference

**Table 4:** Univariate and fitted estimates of covariates included in the time-dependent Cox proportional hazard models on the risk of blood stream infection and mortality, including variables confined by the modified comorbidity index

Model entry	Variable	Univariate HR	*P* Value	Fitted HR	*P* value	Univariate HR	*P* Value	Fitted HR	*P* Value
		Blood stream infection				Mortality			
RRT initiation	AVF*	1.29	0.016	1.37	0.003	0.97	0.745	0.90	0.223***
	TC*	2.29	< 0.001	2.25	< 0.001	1.75	< 0.001	1.48	< 0.001
	NTC*	7.53	< 0.001	6.92	< 0.001	6.20	< 0.001	5.19	< 0.001
	Sex	1.06	NS	–	–	0.98	NS	–	–
	Age 50–59**	1.14	NS	–	–	1.75	0.001	1.65	0.004
	Age 60–69**	1.05	NS	–	–	2.56	< 0.001	2.20	< 0.001
	Age > 70**	1.01	NS	–	–	4.77	< 0.001	3.70	< 0.001
	Heart Failure	1.30	0.003	–	–	2.05	< 0.001	1.34	< 0.001
	AMI	1.20	0.068	–	–	1.92	< 0.001	1.23	0.013
	Vascular	1.13	NS	–	–	1.78	< 0.001	1.25	0.002
	CNS disease	0.96	NS	–	–	1.45	< 0.001	1.14	0.079
	COPD	1.18	0.094	–	–	1.79	< 0.001	1.34	< 0.001
	Collagenoses	0.98	NS	–	–	1.02	NS	0.84	0.122
	Peptic ulcer	1.25	0.039	–	–	1.48	< 0.001	–	–
	Liver disease	1.76	< 0.001	1.52	0.005	1.36	0.028	1.41	0.016
	Cancer	1.18	0.072	–	–	1.81	< 0.001	1.35	< 0.001
	Diabetes	1.34	< 0.001	1.30	< 0.001	1.30	< 0.001	1.22	0.003
Post BSI	AVF*	1.14	0.720	0.98	0.947	0.76	0.252	0.88	0.627 ***
	TC*	2.38	0.025	1.67	0.195	1.25	0.416	1.66	0.086
	NTC*	3.86	0.002	2.36	0.022	3.19	< 0.001	3.58	< 0.001
	Sex	1.13	NS	–	–	1.30	0.037	–	–
	Age 50–59**	1.57	0.096	–	–	1.49	NS	1.70	0.093
	Age 60–69**	1.26	NS	–	–	2.66	< 0.001	2.81	< 0.001
	Age > 70**	1.50	NS	–	–	6.13	< 0.001	6.11	< 0.001
	Heart Failure	1.54	0.020	–	–	1.82	< 0.001	1.43	0.010
	AMI	1.40	NS	–	–	1.80	< 0.001	–	–
	Vascular	1.11	NS	–	–	1.71	< 0.001	–	–
	CNS disease	1.11	NS	–	–	1.43	0.008	–	–
	COPD	1.07	NS	–	–	1.25	NS	–	–
	Collagenosis	1.98	0.003	1.58	0.050	0.78	NS	–	–
	Peptic ulcer	2.00	< 0.001	1.73	0.005	1.43	0.017	–	–
	Liver disease	1.94	0.007	1.82	0.021	1.39	NS	2.23	< 0.001
	Cancer	1.62	0.009	1.36	0.115	2.30	< 0.001	1.64	< 0.001
	Diabetes	1.44	0.019	1.46	0.024	0.97	NS	–	–
	Gram pos	0.84	NS	–	–	0.68	0.003	0.80	0.100

*AMI* acute myocardial infarction, *CNS* central nerve system, *COPD* chronic obstructive pulmonary disease, *HD* hemodialysis, *AVF* arteriovenous fistula, *TC* tunneled catheter, *NTC* non-tunneled catheter, *NS* not significant, *HR* hazard ratio, *CI* confidence interval

*Analyzed as a four-leveled variable with peritoneal dialysis as reference

**Analyzed as a four-leveled variable with ‘age < 50 years’ as reference

***Included in the model as the overall *P*-value for the four-leveled variable was < 0.15

- did not meet the significance levels for final assessments in the fitted models

## Discussion

In the current register-based retrospective cohort study, we investigated the incidence and risk of BSI including the causal microbial species, as well as the risk of all-cause mortality with and without consideration of first-time BSI, in patients receiving PD and HD, respectively, with explicit focus on HD access modality and potential causal risk factors defined by a modified comorbidity index. Gram positive bacteria were the most prevalent causal microorganisms among patients with first-time BSI in the total study population. However, while *S. aureus* dominated the isolates from HD patients, *E. coli* was more frequently isolated in patients on PD. Also, we observed an increasing growth of putative antibiotic-resistant, biofilm producing microorganisms among patients with more than one BSI episode. There was an increased risk of BSI associated with HD as compared to PD, and the highest risks were associated with catheter-based vascular access modalities, i.e., NTC and TC, listed in order of priority. Furthermore, the highest risk of all-cause mortality was associated with NTC followed by TC, and there was no difference between PD and AVF. The overall risk death after first-time BSI was markedly increased as compared to patients without BSI, however most importantly, while the risk of death and BSI remained clearly increased with NTCs in all conducted analyses, there was no significant difference in risk of post BSI mortality between AVF and TC relative to PD, although the trend towards a better outcome for patients with AVF remained. These findings were supported by the absence of difference between the PD and AVF in risk of subsequent BSI episodes, a significant increased risk associated with TC relative to AVF for both outcomes after first-time BSI, as well as a reduced risk of post BSI mortality in patients who changed their vascular access to AVF. Finally, older age was a significant predictor of post BSI mortality but not for subsequent BSI, and the modified comorbidity index was well correlated with both outcomes, although only some of the individual comorbidities confined by the applied comorbidity index were found to be significant in the fitted statistical model. To our knowledge, we are the first to investigate the risk and risk factors of subsequent BSI and post BSI mortality among the different types of vascular access modalities in relation to PD.

Numerous of studies have investigated the epidemiology of BSI, and as supported by the current study, it is generally recognized, that the risk of BSI is markedly increased in HD patients relative to PD, although the overall infection-related hospitalization may be higher with the latter [7, 9–13]. Also, the vascular access type has a major impact on risk of BSI, in which the catheter-based modalities, and in particular NTCs, are found with the highest risk [14, 15]. Although there has been a preferential focus in *S. aureus* bacteremia, the microbiologic fauna in first-time BSIs is comprehensively studied, and the majority of these investigations are supported by our findings, with most isolates being Gram positive (approximate 60%), *S. aureus* being the most frequently isolated causal microorganism, and a relative higher incidence of *E. coli* bacteremia among PD patients [5, 12, 15]. It is also evident, that the risk of all-cause mortality is increased with catheter-based HD modalities; however, as suggested by others [24], we found no significant difference between PD and AVF in risk of all-cause mortality. In fact, there was a trend towards a reduced mortality risk with the latter; and patients who changed their vascular access to AVF after a first-time BSI had a reduced risk of death. Accordingly, as the risk of BSI was higher with AVF relative to PD, these findings suggest that the risk of death among patients with an AVF is less influenced by BSIs as compared to catheter-based RRT modalities, including PD. To our knowledge, there are no data available to differentiate between the specific causes of post BSI mortality with AVF and PD. However, the risk of other dialysis-related infections and subsequent cardiovascular death may be increased in patients on PD, and the risk of metastatic infections in *S. aureus* bacteremia, e.g., endocarditis, osteomyelitis, CNS abscess etc., with subsequent death is markedly increased with TCs [11, 17, 25, 26]. Consequently, in addition to the relatively low IRs of BSI, AVF may be a favorable choice due to an overall better prognosis after BSI.

Although the trend towards an incremental relationship between the included dialysis modalities as well as vascular access types on risk of BSI and death remains in the post BSI population, only patients treated with NTCs were significantly associated with an increased risk of post BSI mortality and subsequent BSI episodes. This could arguably be explained by lack of statistical power judged by the relatively wide confidence intervals. However, it appears plausible that BSI itself temporarily outweighs other risk factors of death and infection associated with dialysis and vascular access, which would explain the lack of difference between AVF and TC relative to PD. In this regard, a previous study showed a comparable 30-day survival after BSI between patients receiving HD and a matched group of population controls, notwithstanding that the risk of BSI was 25 times greater with the HD patients [5]. Interestingly, the otherwise markedly increased risk of death and infection associated with NTCs somewhat declined in the post BSI analyses. NTCs are mainly used use for a shorter time and typically in acute settings, which indeed has influenced on the risk of both death and infection. This is supported by our baseline analyses where a 60-day qualifying period reduced the overall risk of mortality and BSI, hereby highlighting a significant increased risk of these outcomes in patients initiating acute dialyses. However, due to the relatively short usage time of NTCs and as these catheters usually are renewed or removed in favor of other more sustainable access types [27], NTCs may predict access change or renewal in the setting of an ongoing BSI, which is favorable in treatment of a severe catheter-related infections, in which case antibiotics alone seem to be inferior [28]. Conversely, one may be more reluctant to remove TCs as compared to NCTs, which may increase the risk of subsequent infections [29, 30], especially with antibiotic resistant and biofilm-producing microorganisms which, in the current study, were more frequently isolated in patients with more than two episodes of BSI. In this regard, TCs were also associated with an increased risk of post BSI mortality and subsequent BSI as compared to AVF. Finally, there was a trend towards an overall better outcome with AVF in terms of post BSI mortality, and the risk of subsequent BSIs was comparable to that of PD. This was further supported by a significantly better outcome among patients who changed their vascular access to an AVF after first time BSI. Consequently, these results may in part explain the lack of difference between PD and AVF on all-cause mortality, notwithstanding an increased risk of first-time BSI with the latter, as an increased risk of first-time BSI associated with AVF relative to PD, putatively is counter balanced by an actual reduced risk of subsequent BSI and death with AVF, as compared to catheter-based solutions including PD.

While the vascular access type was the only predictor of first-time BSI in our main analysis, advanced comorbidity was additionally associated with subsequent BSI. In this regard, as the risk estimates associated with vascular access types tends to be weakened in context of a recent BSI, it appears plausible that the effect of other risk predictors reach a higher statistical weight in the model. These considerations may also apply for the fitted statistical model, in which all the included elements of the modified comorbidity index were assessed, and where only diabetes and liver disease came out significant in the analyses of first-time BSI; whereas collagenoses, peptic ulcer, liver disease and diabetes were found correlated with increased risk of subsequent BSI. To our knowledge, risk factors associated with subsequent BSI have not previously been investigated; however, there are several studies to support the association between diabetes [8, 12, 15, 31–33] and low plasma albumin [12, 15, 31, 34–39], and the risk of infections, in which the latter risk factor in part may be interpreted as a surrogate marker of liver disease [40, 41]. Also, in concordance with previous findings, advanced comorbidity and age was associated with all-cause mortality; and as a novel finding, this association remained significant after first-time BSI. However, while all the analyzed possible risk predictors except for sex were significantly associated with all-cause mortality in the fitted statistical model, only age, heart failure, liver disease, and cancer was associated with an increased risk of post BSI mortality. There are several causal explanations for death in a cohort of patients on dialysis apart from BSI, in which cardiovascular disease are the most frequent [3]. Also, the applied comorbidity index was based on CCI, which originally was verified to predict the one-year mortality in patients with several co-occurring comorbidities [42]. Accordingly, we expected the included comorbidities to be associated with all-cause mortality in the fitted model, whereas, it appears plausible that only some of these came out significant in analyses of post BSI death, as BSI itself is strongly correlated with death and other potential risk factors may not contribute further to the equation.

## Conclusions

The current study presents novel data on the risk of subsequent BSI and post BSI mortality to support a decline in difference between PD and HD vascular access modalities in the period after first-time BSI; however, there was a persistently marked increased risk of both outcomes with NTC. Furthermore, we suggest that patients with an AVF have a favorable prognosis after first-time BSI in terms post BSI mortality and subsequent BSI in relation to any other analyzed vascular access type, and that AVF at least is as safe as PD. Finally, although a CCI-based comorbidity index may predict the risk of subsequent BSI as well as post BSI mortality among dialysis patients, only some of the variables contained in the index were found to be successful risk predictors of these outcomes when analyzed in a fitted statistical model. Further studies on the risk and risk factors of post BSI mortality with explicit focus on RRT and vascular access type are warranted.

## Limitations

Despite adjustment for some but not all factors known to influence risk of BSI and mortality among patients receiving dialysis, residual confounding is inherent to observational studies and epidemiological associations do not per se represent causal relationships. Furthermore, results might have been affected by limited sensitivity of the diagnostic codes by which these risk factors were defined, and confounding may have been introduced by the unequal distribution of baseline parameters, although most of these were accounted for in the Cox analyses. In this regard, one central limitation to the current study was the lack of information regarding the specific infectious focus preceding a positive blood culture, as not all the included BSIs were caused by dialysis related attributes. Hence, some BSIs may have been initiated by infections (e.g. pneumonia or urinary tract infections), which were not directly associated with a specific vascular access or dialysis modality, however, may have been driven by traits, such as advanced comorbidity or frailty, not accounted for in the statistical models. Nonetheless, confounding introduced by this is putatively negligible, as sensitivity analyses in the current study that exclusively included BSIs caused by *S. aureus*, which is the most frequently isolated microorganism in blood cultures from patients receiving HD, consistently confirmed our main results. Moreover, as the risk of BSI recently was found to be 28 times greater in patients receiving HD than in a matched population without kidney disease [5], most of the BSIs among patients receiving HD is putatively related to their vascular access and frequent breaches of the natural skin barrier. Changes from one blood access modality to another were derived from the National Patient Registry, which contains data of all operations. Another major limitation in this study was that while registration of AVFs putatively is exhaustive as the respective surgical departments in part are financed through their listing of procedure codes, registration of change from one catheter type to another may have been underreported, as this often is done in an acute setting, especially regarding NTCs. Nonetheless, we tried to correct for this putative lack of registration by censoring patients who were registered with a NTC for more than 3 months, which in the worst case would underestimate the risk of NTCs.

## Additional files

Additional file 1: Isolates from the 13 major microbial groups applied in Table 1. Table showing all isolated microorganisms applied in the study. (DOCX 15 kb)

Additional file 2: Baseline analyses on risk of BSI and all-cause mortality/post BSI mortality at index date, index date plus 60 days, and after first-time BSI. Table showing additional results from the baseline analyses on risk of BSI and all-cause mortality/post BSI mortality at index date, index date plus 60 days, and after first-time BSI. (DOCX 22 kb)

## Abbreviations

AVF: arteriovenous fistula
AVG: arteriovenous graft
BSI: blood stream infection
CIN: chronic interstitial nephropathy
CKD: chronic kidney disease
CNS: central nerve system
COPD: chronic obstructive pulmonary disease
DM: diabetes mellitus
DNR: the Danish Nephrology Registry
GN: glomerulonephritis
HD: hemodialysis
HR: hazard ratio
ICD: international classification of disease
IR: incidence rate
MCCI: modified Charlson comorbidity index
MiBa: the Danish Microbiology Database
NCSP: Nordic Classification of surgery Procedures
NTC: non-tunneled catheter
PD: peritoneal dialysis
RRT: renal replacement therapy
TC: tunneled catheter
