# Supplementary material for: Risk of death after first-time blood stream infection in incident dialysis patients with specific consideration on vascular access and comorbidity
Source: BMC Infect Dis. 2018 Dec 20;18:688. doi: 10.1186/s12879-018-3594-7 (PMC6302499; doi:10.1186/s12879-018-3594-7)
Supplement: Supplementary file 1 — Isolates from the 13 major microbial groups applied in Table 1. Table showing all isolated microorganisms applied in the study. (DOCX 15 kb) [file 12879_2018_3594_MOESM1_ESM.docx]

| **Additional file 1. Isolates from the 13 major microbial groups applied in table 1.** | | | | | | |
| --- | --- | --- | --- | --- | --- | --- |
| Enterococci | Enterobacter | Other gram-negative | Other gram-positive | CNS | Streptococci | Fungi |
| Enterococcus faecalis | Enterobacter  species* | Achromobacter xylosoxidans | Bacillus species* | Staphylococcus  capitis | Streptococcus alactolyticus | Candida albicans |
| Enterococcus  faecium | Enterobacter cloacae | Acinetobacter species* | Clostridium species* | Staphylococcus  epidermidis | Streptococcus constellatus | Candida glabrata |
|  | Enterobacter avium | Aeromonas punctate | Corynebacterium jeikeium** | Staphylococcus  lugdunensis | Streptococcus  oralis | Candida tropicalis |
|  | Leclercia adecarboxylata | Bacteroides species | Kocuria kristinae |  | Streptococcus pneumoniae |  |
|  | Yersinia enterocolitica | Citrobacter species* | Lactobacillus species* |  | Streptococcus salivarius |  |
|  | Yersinia pseudotuberculosis | Fusobacterium nucleatum | Listeria monocytogenes |  | Beta-hemolytic  Gr A |  |
|  |  | Haemophilus influenzae | Micrococcus luteus |  | Beta-hemolytic  Gr B |  |
|  |  | Morganella morganii | Micrococcus species* |  | Beta-hemolytic  Gr C |  |
|  |  | Neisseria meningitidis | Propionibacterium acnes |  | Beta-hemolytic  Gr G |  |
|  |  | Proteus species* | Propionibacterium species |  | Non-hemolytic |  |
|  |  | Raoultella ornithinolytica | Rhodococcus species* |  |  |  |
|  |  | Salmonella species* |  |  |  |  |
|  |  | Serratia marcescens |  |  |  |  |
| The following groups, *S. aureus*, *E. coli*, *Klebsiella pneumoniae* (*klebsiella*)*,* and *Pseudomonas aeuroginosa* (*pseudomonas*) only included the selected microorganisms denominated by the group names, respectively. In the group of Unspecified Staphylococci, only the genus (staphylococci) was reported, and not the species. Finally, the group Other only included positive blood cultivations reported without genus or species.  Gr: group; CNS: Coagulase Negative Staphylococci.  *Microorganisms classified within this genus | | | | | | |
