# Supplementary material for: Risk of death after first-time blood stream infection in incident dialysis patients with specific consideration on vascular access and comorbidity
Source: BMC Infect Dis. 2018 Dec 20;18:688. doi: 10.1186/s12879-018-3594-7 (PMC6302499; doi:10.1186/s12879-018-3594-7)
Supplement: Supplementary file 2 — Baseline analyses on risk of BSI and all-cause mortality/post BSI mortality at index date, index date plus 60 days, and after first-time BSI. Table showing additional results from the baseline analyses on risk of BSI and all-cause mortality/post BSI mortality at index date, index date plus 60 days, and after first-time BSI. (DOCX 22 kb) [file 12879_2018_3594_MOESM2_ESM.docx]

|  |  |  |  |  |  |
| --- | --- | --- | --- | --- | --- |
| **Additional file 2. Baseline analyses on risk of BSI and all-cause mortality/post BSI mortality at index date, index date plus 60 days, and after first-time BSI** | | | | | |
| Model entry | Modality/Access | HR (CI) | P value | HR (CI) | P value |
|  |  | **Blood stream infection** |  | **Mortality** |  |
| RRT initiation | PD | 1 | - | 1 | - |
|  | - AVF | 2.68  (1.92-3.74) | <0.001 | 1.13  (0.90-1.43) | NS |
|  | - TC | 5.33  (3.80-7.48) | <0.001 | 3.23  (2.60-4.02) | <0.001 |
|  | - NTC | 13.2  (9.32-18.7) | <0.001 | 10.5  (8.15-13.5) | <0.001 |
|  |  |  |  |  |  |
|  |  |  |  |  |  |
| RRT initiation +60 days | PD | 1 | - | 1 | - |
|  | - AVF | 2.56  (1.77-3.69) | <0.001 | 1.15  (0.90-1.47) | NS |
|  | - TC | 3.69  (2.46-5.53) | <0.001 | 2.60  (2.03-3.31) | <0.001 |
|  | - NTC | 8.68  (5.89-12.8) | <0.001 | 6.44  (5.09-8.15) | <0.001 |
|  |  |  |  |  |  |
| Post BSI | PD | 1 | - | 1 | - |
|  | - AVF | 1.15  (0.53-2.49) | NS | 1.36  (0.73-2.54) | NS |
|  | - TC | 2.52  (1.18-5.37) | 0.017 | 2.12  (1.13-3.97) | 0.019 |
|  | - NTC | 5.19  (2.38-11.3) | 0.020 | 3.85  (2.06-7.18) | <0.001 |
|  |  |  |  |  |  |
| Post BSI *  (*S. aureus*) | PD | 1 | - | 1 |  |
|  | - AVF | 0.60  (0.15-2.37) | NS | 0.55  (0.15-2.02) | NS |
|  | - TC | 1.00  (0.26-3.87) | NS | 0.93  (0.26-3.45) | NS |
|  | - NTC | 1.55  (0.39-6.15) | NS | 1.92  (0.54-6.78) | NS |
| HR: hazard ratio; CI: confidence interval; PD: peritoneal dialysis; AVF: arteriovenous fistula; TC: tunneled catheter; NTC: non-tunneled catheter; BSI: blood stream infection; NS: not significant.  *Baseline subgroup analysis of patients with a first-time BSI caused by S*. aureus* | | | | | |
